# Barriers to substance use and mental health utilization among Asian-American women: exploring the conflict between emotional distress and cultural stigma

**DOI:** 10.1186/1940-0640-10-S1-A2

**Published:** 2015-02-20

**Authors:** Astraea Augsberger, Hyeouk Chris Hahm, Albert Yeung, Meaghan Dougher

**Affiliations:** 1 School of Social Work, Boston University, Boston, MA, 02215, USA; 2 Department of Psychiatry, Harvard University, Cambridge, MA, 02138, USA

## Background

There has been growing incidence of depression, suicide, substance use, and sexually transmitted infections among Asian-American women. However, they are reluctant to seek health and mental health services. There is scant empirical research focused on potential barriers to health-care utilization among Asian-American women. The present paper contributes to the literature by providing an indepth exploration of the factors Asian-American women identify as preventing them from seeking health-care services.

## Materials and methods

The qualitative interviews utilized in this analysis are part of a mixed-methods study, the Asian-American Women’s Sexual Health Initiative Project (AWSHIP), designed to examine health behaviors amongst Chinese, Korean, and Vietnamese women aged 18–35, who are children of immigrants. Thirty-six participants were interviewed using a semi-structured interview guide consisting of questions related to substance use, mental health, and health-care utilization. All interviews were audio recorded and transcribed verbatim. Data were analyzed using thematic analysis, including a cursory review of the data, developing initial codes, applying initial codes to additional data, expanding upon the codes, collating the codes into potential themes, and building a thematic map [1].

## Results

Participants felt isolated and alone when handling substance use and mental health concerns. Participants identified cognitive factors such as shame and stigma as important issues permeating the Asian-American mentality on health care. For example, participants emphasized the importance of being perceived by the Asian community as strong. Disclosing a mental health issue was perceived as being weak. Participants were confronted with the conflict between expressing their own emotional distress and the cultural stigma associated with disclosing emotional vulnerabilities. Participants also identified practical factors, including services being perceived as unhelpful in addressing their concerns, professionals lacking a cultural understanding, and financial concerns. While some participants benefitted from psychotropic medication, others perceived taking medication as being weak.

## Conclusions

Both cultural and practical considerations were discussed as barriers for access to mental health care utilization among Asian-American women. Shame and stigma were found to be dominant cultural barriers, even though all participants in the study are children of immigrants, who were either born in the United States or grew up in the United States since childhood. A lack of mental health professionals who can truly understand their culturally specific needs was identified as a major practical barrier. In order to address the alarming problem of substance use and mental health issues among this population, efforts should be targeted towards reducing the shame and stigma in health-care utilization and creating culturally specific interventions for Asian-American women.
